# S100B, Homocysteine, Vitamin B12, Folic Acid, and Procalcitonin Serum Levels in Remitters to Electroconvulsive Therapy: A Pilot Study

**DOI:** 10.1155/2018/2358451

**Published:** 2018-01-10

**Authors:** Hannah Maier, Saskia Helm, Sermin Toto, Nicole Moschny, Wolfgang Sperling, Thomas Hillemacher, Kai G. Kahl, Ewgeni Jakubovski, Stefan Bleich, Helge Frieling, Alexandra Neyazi

**Affiliations:** ^1^Department of Psychiatry, Social Psychiatry and Psychotherapy, Hannover Medical School, Hannover, Germany; ^2^Center for Systems Neuroscience, Hannover, Germany; ^3^Department of Psychiatry and Psychotherapy, Friedrich-Alexander-University Erlangen-Nürnberg, Erlangen, Germany

## Abstract

**Background:**

Electroconvulsive therapy (ECT) is one of the most effective treatment options for refractory depressed patients. To date, there are only a few predictors of response.

**Aim:**

The aim was to identify predictive biomarkers of remission to ECT on a molecular level.

**Methods:**

11 patients suffering from a major depressive episode—according to the Statistical Manual of Mental Disorders, Fourth Edition (DSM-IV)—underwent 10 ECT sessions. Blood samples were taken, and the depression severity was assessed before, one hour and 24 hours after sessions 1, 4, 7, and 10 using the Montgomery Asberg Depression Rating Scale (MADRS). A MADRS total score < 12 was interpreted as remission.

**Results:**

Patients remitting under ECT had significantly higher homocysteine (*p* < 0.001), S100B (*p* < 0.001), and procalcitonin (PCT) (*p* = 0.027) serum levels. On the contrary, serum levels of vitamin B12 (*p* < 0.001) and folic acid (*p* = 0.007) were significantly lower in remitters compared to those in nonremitters. Levels remained unchanged throughout the whole ECT course.

**Conclusions:**

Our findings indicate that lower levels of vitamin B12 and folic acid associated with higher levels of homocysteine, S100B, and PCT point to a subgroup of depressed patients sensitive to ECT. Due to the limited sample size, further studies are required to replicate our findings.

## 1. Introduction

Electroconvulsive therapy (ECT) is one of the most effective treatment options in refractory depressed patients [1]. However, not all patients benefit from ECT and only a few predictors of response are established for routine clinical use.

A meta-analysis of Haq and colleagues revealed that longer depressive episodes and medication failure at baseline are reliable predictors of poor ECT response. The number of previous depressive episodes, gender, or age at onset did not predict ECT treatment outcome. Other studies reported higher rates of remission in elderly patients, though it still remains unclear whether age could be a useful predictor independent of medication failure and duration of the current depressive episode [2].

Besides the patient's individual characteristics, the cardiovascular reaction is known to be associated with response to ECT: elevated postictal physiological parameters—such as diastolic and systolic blood pressure and heart rate—indicate high ECT efficacy [3]. Furthermore, the length of the motoric seizure (>20 sec), the seizure seen in the electroencephalogram (EEG) (>25 sec), the postictal suppression index (>80%), the synchronicity of the hemispheres (>90%), and the height of the amplitudes (>180 *μ*V) are indicating the seizure quality for high ECT efficacy [4–8]. Finally, the speed of response to ECT has been reported to have a predictive value concerning ECT remission [9]. Within the last years, an increasing number of studies investigated biomarkers for the prediction of the response to antidepressant treatments, while only a few looked into biomarkers in ECT.

It is well known that a lack of some vitamins like vitamin B12 or folic acid can lead to neuropsychiatric syndromes such as depression or dementia [10–13]. Concerning major depressive disorder (MDD), patients with increased vitamin B12 serum levels are more likely to respond to antidepressants than patients with low levels of vitamin B12 [10, 14]. Furthermore, Fava and colleagues stated that low folate levels may be linked with inferior antidepressant treatment outcome concerning fluoxetine, a serotonin reuptake inhibitor (SSRI) [15]. Folic acid and vitamin B12 deficiency might be—among other possibilities—a result of long-term antidepressant treatment: Labadarios and colleagues hypothesized that folic acid is needed for de novo synthesis of microsomal enzymes and used as a cofactor for methylation reactions and hydroxylations. Indeed, many drugs are metabolized through the mixed function oxidase system and are therefore likely to cause folate depletion. Labadarios and colleagues reported slightly lower serum and blood cell folate concentrations in patients taking tricyclic antidepressants compared to a control group. They did not find any changes concerning vitamin B12 levels [16]. Farrell and colleagues on the other hand could not replicate the results relating to decreased folic acid level antidepressant ingestion ten years later. They assumed that different measurement methods or a too short observation time in the latter study was the cause of the difference [17]. Nevertheless, a supplementation with vitamin B12 or folic acid in depressive disorders is not generally recommended according to a meta-analysis by Almeida and colleagues [18].

Vitamin B12 and folic acid are needed for the methylation and depletion of homocysteine [19]. Elevated homocysteine levels, hyperhomocysteinemia, increase the risk for metabolic syndrome and lead to endothelial dysfunction causing atherosclerosis as well as microinflammation [20, 21]. In fact, markers of inflammation, for example, CRP, are also associated with depressive disorders [22]; however, the effects of ECT to acute phase reactants such as CRP and PCT are not well known yet. Giltay and colleagues reported an increase of PCT during ECT treatment, whereas CRP levels remained unchanged in their study [23–25]. Further, hyperhomocysteinemia has been associated with the development of certain somatic and psychiatric diseases, for example, major depressive disorder or dementia [26, 27]. Since homocysteine influences DNA methylation, epigenetic changes in MDD have been proposed to be related to homocysteine levels [28]. Nevertheless, there is little known concerning homocysteine and ECT. Regarding seizures during alcohol withdrawal, our group proposed an association between hyperhomocysteinemia and an overstimulation of the N-methyl-D-aspartate (NMDA) receptor [29]. Interestingly enough, our group found no relationship between elevated homocysteine levels, ECT seizure duration, and changes in ECT efficacy [30]. By activating the NMDA glutamate receptor [31], increasing the blood-brain barrier permeability, and elevating oxidative stress [32], hyperhomocysteinemia impairs the function of dopaminergic [33], cerebellar Purkinje neurons [34] and astrocytes in vitro [35] and thus causes neuronal damage. A known marker of neuronal damage is S100B, a glial calcium-binding protein with neuroplastic properties. Several studies emphasize the importance of S100B for major depressive disorders: in males with minor depressive episodes, S100B levels are increased when compared to healthy subjects [36]. Additional evidence is given by a meta-analysis of Schroeter and colleagues: they reported that serum levels of S100B were consistently increased during acute major depressive episodes and slightly decreased after treatment with antidepressants [37]. Further studies revealed that patients with MDD and higher S100B serum levels have a better therapeutic response to antidepressants compared to those with normal S100B serum levels [38]. Several clinical studies showed no significant correlation between ECT, S100B, and cognitive side effects [39–41]. Palmio and colleagues replicated these findings and were able to show that a transient increase in S100B levels correlated with a reduction of BDI scores. They interpreted their findings, in line with other studies, as a sign for glial cell activation. This activation is suggested to be a main contributor for the antidepressant effect of ECT [42, 43]. Solely, one clinical study measured a small but significant elevation of S100B one hour after ECT administration associated with poorer memory function [44].

The aim of our study was to analyze vitamin B12, folic acid, homocysteine, procalcitonin, and S100B levels in patients undergoing ECT and to compare remitters and nonremitters to the treatment.

## 2. Methods

### 2.1. Patients and Treatment Procedures

We conducted a prospective study of ECT remission in treatment-resistant MDD. Treatment resistance was defined as being nonresponsive to at least two state-of-the-art antidepressant treatments with different substance classes. Diagnoses were established using the German version of the Structured Clinical Interview for Diagnostic (SKID) and Statistical Manual of Mental Disorders, Fourth Edition (DSM IV). Depression severity was assessed before, one hour and 24 hours after ECT treatment in sessions 1, 4, 7, and 10 using the Montgomery Asberg Depression Scale (MADRS), as well as the German version of Beck's Depression Inventory (BDI-II) [45, 46]. A MADRS total score < 12 was interpreted as remission and was the primary outcome variable. ECT was administered as commonly practiced in the facility with a customized Thymatron IV brief-pulse device (Somatics; Lake Bluff, IL, USA). Motor and electroencephalogram seizure duration was monitored, and stimulus intensity was adjusted accordingly. Three ECT sessions per week were applied over three and a half weeks. Study details have been reported in detail elsewhere [30, 47]. The study adhered to the Declaration of Helsinki (1964) and its later amendments. It was approved by the Ethics Committee of the University of Erlangen. Written informed consent was obtained from all patients prior to their inclusion into the study and after the procedures had been fully explained to them. All patients were recruited from an inpatient population at the Department of Psychiatry and Psychotherapy of the University Hospital Erlangen.

### 2.2. S100B, Homocysteine, PCT, Vitamin B12, and Folic Acid Serum Levels

Fasting blood samples were taken directly before (8–10 a.m.), one hour and 24 hours after ECT sessions 1, 4, 7, and 10. All blood samples were stored at −80°C immediately after their collection and centrifugation. S100B serum levels were assessed using electrochemiluminescence immunoassay (ECLIA; Cobase 411); PCT serum levels were measured using the TRACE-Technology (Time-resolved Amplified Cryptate Emission; Kryptor). Homocysteine, vitamin B12, and folic acid serum levels were assessed using high-performance liquid chromatography (HPLC; Agilent, Santa Clara, Calif) within 30 minutes after each ECT (15 min, 20 degrees, 2900 rpm) and via electroluminescent devices (E170, Roche, Basel, Switzerland).

### 2.3. Statistical Analysis

Correlation analysis was performed for baseline psychometric data (BDI, MADRS), demographic parameters (body mass index (BMI), age, and gender), and serum levels (homocysteine, vitamin B12, folic acid, S100B, PCT). For normally distributed parameters (Kolmogorov–Smirnov test), Pearson's test was used, and for not normally distributed data, Spearman's rho test was used. Analysis of vitamin B12, folic acid, homocysteine, S100B, or PCT and remission/changes over time under ECT were performed using mixed linear models (fixed factors and their interaction: ECT number, measurement number, and remission). The variables age and BMI were controlled for in the statistical analysis (as random factors) as they are known to impact the investigated substances. Results are presented as mean ± standard deviation (SD). *p* values of less than 0.05 (two-tailed) were considered to indicate statistical significance. Data was analyzed employing IBM SPSS Statistics for Windows, version 21.0 (Armonk, NY: IBM Corp.). Graph Pad Prism 5 (Graph Pad Inc., San Diego, CA) was used for data presentation.

## 3. Results

### 3.1. Baseline Characteristics

Patients' baseline characteristics are shown in Table 1. Four patients remitted to ECT. The average age of the sample was 47 years (SD ± 16.5). There were five women and 6 men included in the study. The average baseline BDI was a score of 36 (SD ± 10.2), and the average baseline MADRS was a score of 34 (SD ± 8.3). There was no significant difference between remitters and nonremitters regarding the baseline characteristics (Fisher's test and Mann–Whitney *U* test: all *p* > 0.100).

### 3.2. Serum Levels of Vitamin B12, Folic Acid, PCT, Homocysteine, S100B, and Depression Severity

Vitamin B12 and folic acid serum levels positively correlated with each other (*p* < 0.001, *r* = 0.58). Vitamin B12 and folic acid negatively correlated with homocysteine and age (vitamin B12 and homocysteine: *p* < 0.001, *r* = −0.38; folic acid and homocysteine: *p* < 0.001, *r* = −0.65; vitamin B12 and age: *p* < 0.001, *r* = −0.36; and folic acid and age: *p* < 0.001, *r* = −0.34). There was no significant correlation between vitamin B12, folic acid, and BMI, as well as PCT. Homocysteine levels positively correlated with S100B and PCT levels (S100B: *p* < 0.001, *r* = 0.48; PCT: *p* = 0.001, *r* = 0.31). Furthermore, homocysteine positively correlated with age (*p* = 0.012, *r* = 0.25) but there was no significant correlation between homocysteine and BMI (*p* = 0.652, *r* = −0.44). Additionally, there was a significant positive correlation between S100B and PCT levels (*p* = 0.004, *r* = 0.28), while S100B did not show any correlation with levels of vitamin B12 or folic acid (S100B and vitamin B12: *p* = 0.81, *r* = −0.24; S100B and folic acid: *p* = 0.128, *r* = −0.16).

The severity of depression was measured with BDI and MADRS. Vitamin B12, folic acid, and homocysteine levels at baseline did not correlate with the severity of depression in neither of the tests. Both scales negatively correlated with baseline S100B levels (S100B and BDI: *p* < 0.001, *r* = −0.5; S100B and MADRS: *p* = 0.001, *r* = −0.41). Additionally, a negative correlation between PCT and MADRS was found (*p* = 0.030, *r* = −0.26).

### 3.3. Remission and Serum Levels of Vitamin B12, Folic Acid, PCT, Homocysteine, and S100B

**Figure 1:**
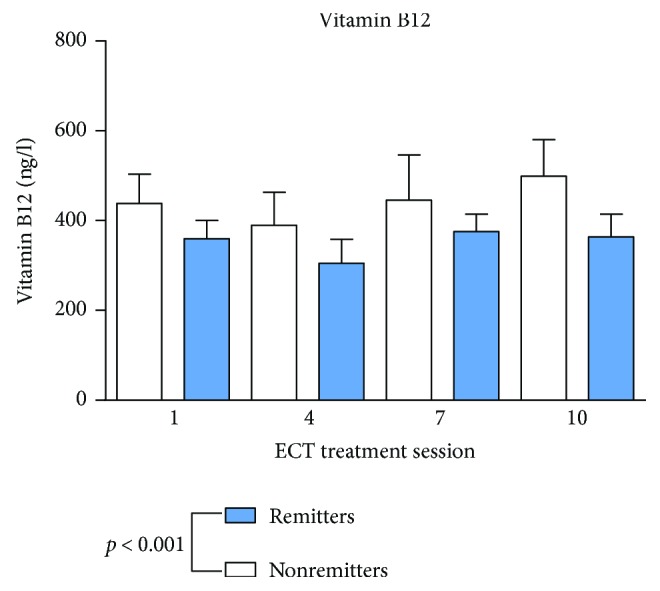
Vitamin B12 serum levels in remitters and nonremitters. Mixed linear modelling showed significantly lower levels of vitamin B12 in remitted patients. The *p* value given in the figure is derived from mixed linear modelling. Error bars show the standard error of the mean (SEM).

**Figure 2:**
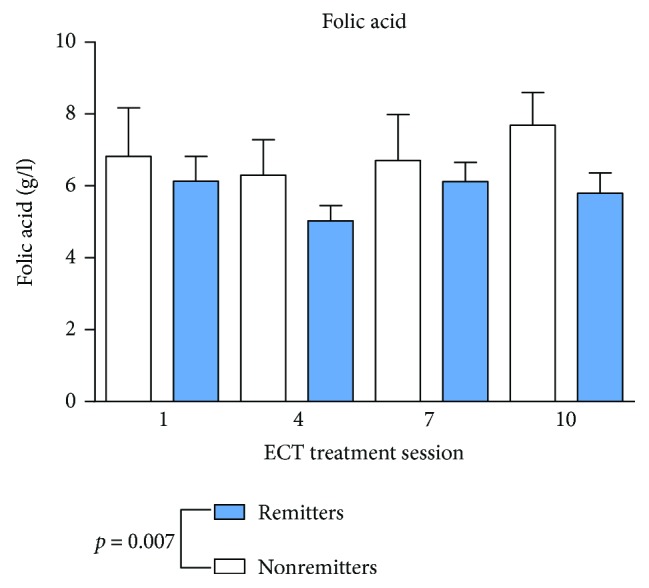
Folic acid serum levels in remitters and nonremitters. Mixed linear modelling showed significantly lower serum levels of folic acid in remitted patients. The *p* value given in the figure is derived from mixed linear modelling. Error bars show the standard error of the mean (SEM).

**Figure 3:**
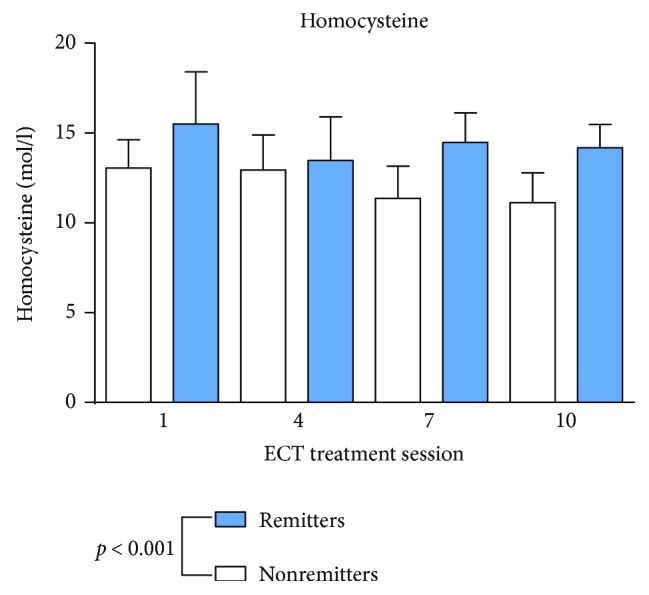
Homocysteine serum levels in remitters and nonremitters. Mixed linear modelling showed significantly higher serum levels in homocysteine in remitted patients. The *p* value given in the figure is derived from mixed linear modelling. Error bars show the standard error of the mean (SEM).

**Figure 4:**
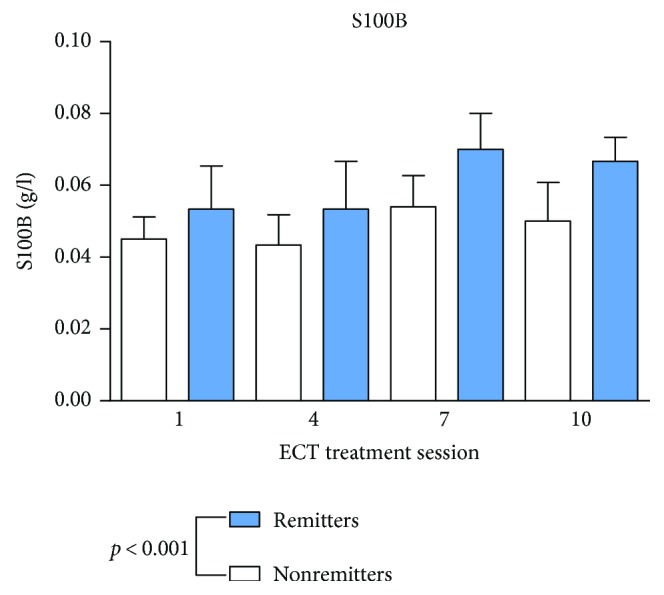
S100B serum levels in remitters and nonremitters. Mixed linear modelling showed significantly higher serum levels of S100B in remitted patients. The *p* value given in the figure is derived from mixed linear modelling. Error bars show the standard error of the mean (SEM).

Remission was defined as MADRS < 12 according to the prolonged, chronic, and treatment-resistant MDD [48]. Standard values and mean values are shown in Table 2. As shown in Figures 1 and 2, vitamin B12 and folic acid showed lower serum levels in remitters to ECT at baseline measurements and through the whole ECT course (vitamin B12: *p* < 0.001; folic acid: *p* = 0.007) compared to nonremitters. There was no significant change during the assessments before, one hour or 24 hours after ECT (vitamin B12: *p* = 0.818; folic acid: *p* = 0.759) or during the ECT series (vitamin B12 *p* = 0.526; folic acid *p* = 0.476). Homocysteine (Figure 3) showed higher baseline serum levels in remitters to ECT (homocysteine: *p* < 0.001), except in the fourth ECT. S100B (Figure 4) and PCT (data not shown) showed higher baseline serum levels in remitters to ECT (S100B:*p* < 0.001; PCT: *p* = 0.027), but there was no significant change in homocysteine, S100B, and PCT levels when measured before, one hour or 24 hours after ECT (homocysteine: *p* = 0.817; S100B: *p* = 0.763; and PCT: *p* = 0.209) or during the ECT series (homocysteine: *p* = 0.450; S100B *p* = 0.066; and PCT *p* = 0.783).

## 4. Discussion

The aim of the present study was to investigate the serum levels of S100B, homocysteine, vitamin B12, folic acid, and PCT during a course of ECT.

The major finding of this study is the difference between S100B, homocysteine, vitamin B12, folic acid, and PCT serum levels in remitters to ECT compared to nonremitters: patients remitting under ECT had significantly higher homocysteine, S100B, and PCT serum levels, whereas serum levels of vitamin B12 and folic acid were significantly lower in remitters to ECT than in nonremitters. There was no significant change from pre-ECT to one hour or 24 hours post ECT. These differences were present over the whole ECT time course (in terms of a main effect), but there was no change throughout the intervention (in terms of an interaction). The homocysteine level at the fourth ECT showed no difference in remitters to nonremitters. No reason was found for the alignment of the values. Especially, patients with signs of neuronal damage (reflected by S100B levels at baseline) seem to remit while undergoing ECT. The neuronal damage could be related to the observed higher homocysteine levels. By using a rodent model of chronic unpredictable mild stress, Chengfeng and colleagues demonstrated that hyperhomocysteinemia could be a result of depressive disorders [49]. Our results could also indicate a subtype of MDD with higher homocysteine, S100B, and PCT levels accompanied with lower vitamin B12 and folic acid levels. The higher homocysteine levels can be interpreted as a sign of inflammation and stress [13, 49, 50]. ECT could reduce this stress [2]; therefore, patients with MDD and higher homocysteine levels would be more likely to remit under ECT treatment than other subgroups. The availability of vitamin B12 and folic acid is limited and therefore decreasing while, among other duties, methylating homocysteine which is upregulated during stress [19]. Another possibility for the elevated homocysteine levels could be folic acid depletion through the long-term use of antidepressants [16, 17], though evidence for this hypothesis is very low. Different studies which investigated the supplementation of vitamin B12 and folic acid during depressive episodes showed no significant effects on depressive symptoms [51, 52]. On the other hand, previous studies reported that a low vitamin B12 and folic acid status is associated with poor response to antidepressive treatments [10, 14]. However, there is no sufficient evidence supporting sole vitamin B12 and folic acid supplementation in patients with MDD, although it seems quite reasonable. On the other hand, previous studies reported that a low vitamin B12 and folic acid status is associated with poor response to antidepressive treatments [10, 14]. In this regard, it would be interesting if taking antidepressants influences the vitamin B12 status. There is solely the study of Labadarios and colleagues where they found no changes in the vitamin B12 levels while taking antidepressants [16, 17]. In our study, patients with lower vitamin B12 and folic acid serum levels did remit while undergoing ECT. Since there were no follow up examinations, we were not able to detect any belated adaptations concerning vitamin B12, folic acid, or homocysteine levels as shown by Chengfeng et al. in a rodent model [49]. Even though general supplementation with vitamin B12 and folic acid was not recommended, one could speculate that patients with MDD and higher homocysteine levels could benefit from a supplementation of vitamin B12 and folic acid supplementation while undergoing ECT.

As in previously reported studies, we did not find any differences in S100B levels during the whole course of ECT [39–41]. In line with our findings, Kranaster and colleagues did not find a correlation between S100B serum levels and the completion of an ECT session in terms of glial damage [39]. A mild elevation of S100B levels can be interpreted as neuroprotective since S100B acts as a neurotrophic factor in the developing brain. On the contrary, a release of S100B can lead to neuroinflammation and neuronal dysfunction [53]. Intriguingly, S100B at baseline has been considered as a possible marker for cognition side effects and depression [44]. However, Kranaster and colleagues did not find any correlation between baseline S100B levels and the cognitive decline of ECT patients [39]. Our study supports this result, as the baseline S100B levels were significantly higher in remitters and correlated negatively with baseline depression severity. Another possible explanation for the increased S100B level could be the microinflammation caused by elevated homocysteine levels: increased homocysteine levels and, as a result, excessive oxidative stress and NMDA receptor activation might lead to neuronal impairment following a subsequent release of S100B and PCT. Our data showed a positive correlation between PCT and S100B in combination with elevated homocysteine levels. In a rodent model with intracerebral injection of homocysteine, Kamat and colleagues found an increase of neuronal microinflammation and therefore S100B [54]. These findings are in line with the work of Wedekind and colleagues, who found that both homocysteine and S100B levels can be increased in alcohol withdrawal patients. They interpreted their findings as a possible neuroprotective release since the elevation was mild [55]. Additionally, it has been suggested that serotonergic neurons are able to regulate their own sprouting and regeneration through the release of S100B [56]. Besides neurons, also other cell populations are able to secrete S100B [57–59]: Moutsatsou and colleagues, for example, showed that leukocytes secrete S100B in bipolar disorders, whereas the role of S100B for unipolar depression has not been clarified yet [59].

It still remains elusive how homocysteine increases the S100B serum levels, since in our findings, the elevation of S100B in remitters to ECT was mild and can therefore alternatively be interpreted as a regeneration process.

The main limitation of our study is the small sample size. Additionally, we compared remitted and nonremitted patients with MDD through an ECT course and considered differences and not absolute values for homocysteine, S100B, PCT, vitamin B12, and folic acid. Therefore, our findings must be considered preliminary and replications in larger groups are needed.

We were able to show that remitters to ECT had increasing levels of homocysteine, S100B, and PCT as well as lower levels of vitamin B12 and folic acid compared to nonremitters. In conclusion, our findings indicate that a deficit of vitamin B12 and folic acid associated with elevated homocysteine levels and elevated S100B levels could point to a subgroup of patients especially sensitive to ECT and contribute to the search of biomarker sets within the heterogenous syndrome of depressive disorders. These findings may lead to a more specific and individualized treatment of patients. However, more studies concerning possible biomarkers for remission to ECT are needed.

## Figures and Tables

**Table 1:** Baseline characteristics of remitters and nonremitters.

Characteristics	Nonremitters (*n* = 7)	Remitters (*n* = 4)
Age, years, mean (SD)	51 (16.0)	46 (19.3)
Women, *n* (%)	3 (42.9)	2 (50.0)
Body mass index, mean (SD)	28 (2.4)	23 (2.5)
MADRS, mean (SD)	34 (5.8)	32 (12.6)
BDI, mean (SD)	38 (7.9)	28 (13.7)
Duration of current depressive episode, weeks, mean (SD)	29 (33.2)	23 (2.3)
Age at initial diagnosis, years, mean (SD)	40 (11.4)	26 (14.8)
Number of previous depressive episodes, mean (SD)	5 (3.5)	4 (2.3)
Psychotic symptoms, *n* (%)	5 (71)	3 (75)
History of suicide attempt, *n* (%)	2 (29)	0 (0)
Antidepressants, *n* (%)	6 (86)	4 (100)
Atypical antipsychotics, *n* (%)	5 (71)	4 (100)

Fisher's exact test and Mann–Whitney *U* test revealed no significant differences between the groups (all *p* > 0.100). *n*: number; SD: standard deviation.

**Table 2:** Mean values and standard values of measured markers.

Markers	Nonremitters (*n* = 7)	Remitters (*n* = 4)	Standard values
Homocysteine, mean (SD)	11.98 (3.82)	14.20 (3.02)	6–12 *μ*mol/l
Vitamin B12, mean (SD)	417.57 (169.24)	343.57 (64.83)	229–812 pmol/l
Folic acid, mean (SD)	7.46 (5.44)	5.79 (0.99)	3–15 ng/ml
S100*β*, mean (SD)	0.05 (0.05)	0.07 (0.04)	<0.1 *μ*g/l
PCT, mean (SD)	0.06 (0.02)	0.07 (0.04)	<0.5 *μ*g/l

*p* values are given in the text. *n*: number; SD: standard deviation.
